# Age related changes of rib cortical bone matrix and the application to forensic age-at-death estimation

**DOI:** 10.1038/s41598-021-81342-0

**Published:** 2021-01-22

**Authors:** Andrea Bonicelli, Peter Zioupos, Emily Arnold, Keith D. Rogers, Bledar Xhemali, Elena F. Kranioti

**Affiliations:** 1 Edinburgh Unit for Forensic Anthropology, School of History Classics and Archaeology, University of Edinburgh, Edinburgh, UK; 2 Musculoskeletal & Medicolegal Research Group, Cranfield Forensic Institute, Defence Academy of the UK, Shrivenham, UK; 3 Materials Science and Radiation Group, Cranfield Forensic Institute, Defence Academy of the UK, Shrivenham, UK; 4 Institute of Forensic Medicine, Tirana, Albania; 5 Forensic Medicine Unit, Department of Forensic Sciences, Faculty of Medicine, University of Crete, Heraklion, Greece

**Keywords:** Biomaterials, Characterization and analytical techniques

## Abstract

Forensic anthropology includes, amongst other applications, the positive identification of unknown human skeletal remains. The first step in this process is an assessment of the biological profile, that is: sex, age, stature and ancestry. In forensic contexts, age estimation is one of the main challenges in the process of identification. Recently established admissibility criteria are driving researchers towards standardisation of methodological procedures. Despite these changes, experience still plays a central role in anthropological examinations. In order to avoid this issue, age estimation procedures (i) must be presented to the scientific community and published in peer reviewed journals, (ii) accurately explained in terms of procedure and (iii) present clear information about the accuracy of the estimation and possible error rates. In order to fulfil all these requirements, a number of methods based on physiological processes which result in biochemical changes in various tissue structures at the molecular level, such as modifications in DNA-methylation and telomere shortening, racemization of proteins and stable isotopes analysis, have been developed. The current work proposes a new systematic approach in age estimation based on tracing physicochemical and mechanical degeneration of the rib cortical bone matrix. This study used autopsy material from 113 rib specimens. A set of 33 parameters were measured by standard bio-mechanical (nanoindentation and microindentation), physical (TGA/DSC, XRD and FTIR) and histomorphometry (porosity-ImageJ) methods. Stepwise regressions were used to create equations that would produce the best ‘estimates of age at death’ vs real age of the cadavers. Five equations were produced; in the best of cases an equation counting 7 parameters had an R^2^ = 0.863 and mean absolute error of 4.64 years. The present method meets all the admissibility criteria previously described. Furthermore, the method is experience-independent and as such can be performed without previous expert knowledge of forensic anthropology and human anatomy.

## Introduction

Age estimation remains one of the most challenging tasks in establishing the biological profile of unknown skeletal remains. Following the Daubert (United States Supreme Court in Daubert vs Merrell Dow Pharmaceuticals, 1993) ruling on admissibility of expert witness testimony^1,2^, validation of age estimation methods has been a fundamental point of discussion. The main principles chosen by the scientific community in order to accept an age estimation procedure on unidentified human remains can be summarised in three main points^1^. First, the method needs to be presented to the scientific community via peer-reviewed publications. Second, the method must provide clear indication of its accuracy regarding the specific case it is applied to. Finally, the method needs to be accurate and repeatable. However, more aspects need be considered in order to evaluate the applicability of a method. Additionally, assessed features should change consistently in all individuals so that they are applicable to samples other than the initial reference one. Furthermore, the statistical approach chosen should be able to classify data (categorical or continuous) without relying on observer error in order to provide consistency to the results^3^. These requirements are not always easy to meet, thus introducing error in the age estimates produced.

Further, experience remains one of the main factors in any anthropological assessment based on visual estimation. Baccino et al.^4^ evaluated the efficiency of age-at-death estimations based on the Suchey-Brooks method for the pubic symphysis, the İşcan et al. method for the sternal end cartilage calcification of the fourth rib^5^, the single root translucency by Lamendin et al.^6^ and the histomorphometric analysis of the femur developed by Kerley et al.^7^, on a French population. The two observers (one forensic pathologist and one forensic anthropologist) appeared to be able to estimate age without significant difference. Inaccuracy was, however, the highest for the histological method potentially due to the inexperience of the observers. In addition, the pubis symphysis method, although it exhibited relatively low inter-observer inaccuracy, also showed significant differences in terms of observer variability. The article also tested the effectiveness of a multifactorial approach and found that this performs generally better than each single method used in isolation^4^. Similarly, Garvin and Passalacqua^8^ analysed the preferences of 145 forensic anthropologists for age estimation approaches. In terms of method selection, Suchey-Brooks remains the favoured method followed by cranial sutures and dental wear, regardless of practitioner experience level. One interesting outcome of this study was that the vast majority of anthropologists stated that they chose the age estimation methodological approach on a case by case basis^8^. Overall, results indicate that in order to perform morphological age estimation, experience plays a central role, especially at the time of choosing the appropriate method, suggesting that the case itself drives the choice only to a limited extent^8^. This is a non-quantifiable source of bias that cannot be overlook by witness admissibility. Another technical issue relies on the fact that the majority of methods are developed on a specific reference population and this could prevent operators using them on samples of unknown populations. Konigsberg and Frankenberg^9^ cautioned that applying a method to a different population can increase the error rate and this was verified for most traditional methodologies^10–13^. This could also be the case for inter-population variation due to factors such as income, diet and activity^3,14,15^. Endogenous and exogenous factors additionally introduce a high degree of inaccuracy in individuals of advanced age and as a result these methods are vastly unsuitable for forensic applications. However, the issue of ‘age mimicry’ was highlighted early on by Bouquet-Appel and Masset^16^, who stressed the fact that not only the genetic origin of the population can represent a biasing factor but also the composition of the sample used for the development of the methodology^17,18^. Bayesian statistics, and more specifically transition analysis (TA) combined with Bayesian techniques, have been employed to minimize the mimicry effect by employing informative priors to improve prediction. The combination of these two approaches, applied to traditional or newly developed age estimation methods, allows for a transition control between different age stages for biased age estimation to be carried out^19^. Despite the fact that this approach created a large amount of interest due to its potential and has been widely applied^17,18^, the methodology is not free of limitations. For example, if the sample used to create priors does not have the appropriate age profile this could lead to an erroneous estimation. Therefore, the choice between a Bayesian and frequentist approach needs to be case driven to optimise the accuracy of the result^19,20^.

Advances in medical sciences and biomedical engineering have led to the development of several quantitative methodologies for investigating physiological processes related to ageing and pathological conditions. The main advantages offered by these approaches is that they are independent of anthropological experience and provide a highly standardised experimental procedure which is easy to replicate and apply in other contexts. Furthermore, the statistical approach that is involved in these methods produces a measurable degree of error and therefore confidence in the reliability of the estimation^21,22^. Nonetheless, these approaches need validation in order to be applied in forensic settings.

One physiological process largely exploited in forensic anthropology is the racemization of aspartic acid (AAR). It is based on the conversion of optically active amino acids into racemic compounds. All amino acids, with the exception of glycine, have the ability to rotate the plane of plane-polarised light. These optic isomers are known as L-enantiomers (laevorotary) and D-enantiomers (dextrorotary). The change in the ratio of these two isomers, quantified by gas chromatography or high-performance liquid chromatography (HPLC) is related to the natural ageing process with an increase in D-amino acid. Aspartic acid is affected by a high rate of turnover due to its tendency of bonding with acidic residues, resulting in a quick accumulation of D-Asp over L-Asp, making it a suitable target in clinical and forensic settings^21,23^. Teeth have been identified as the most suitable skeletal element for AAR analysis. Both dentin and enamel proteins have a lower turnover rate as compared to bone and it is easy to isolate pathological specimens (e.g., caries) to obtain the best age estimation possible. Caries, for example, have been seen to promote the accumulation of D-aspartic acid resulting in an overestimation of the sample^23,24^. Despite the difficulties in the experimental procedure and the variability of the racemisation process in relation to pH and temperature, it remains significantly more consistent than traditional macroscopic approaches^25^.

Post-translational modifications of collagen have also been identified as a target for age estimation. The physiological accumulation of enzymatically-mediated cross-links^26,27^ evaluated by Martin-De Las Heras et al.^28^ produced an age estimation formula from human molars of 22 individuals that was able to predict age with 65% confidence levels and a mean error of ± 14.9 years. Although the accuracy is not sufficient for admissibility criteria, this could represent a complementary method to apply on teeth and/or an advantageous asset when developing multi-factorial approaches for age estimation.

Recently, both mineral and collagen bone matrices were evaluated by ATR-FTIR in order to assess their potential for age estimation^29^. Eighty human femora and humeri showed that crystallinity and type B carbonate in cortical bone matrix are significantly correlated with age, although correlation is only poor to moderate (R < 0.5). With the advances of imaging technologies, new options for age estimation have emerged. Despite the fact that they normally involve the use of X-rays techniques (e.g., DXA or computed tomography), their main advantage is that they can be carried out non-invasively on both living individuals as well as human remains. Navega et al.^32^ proposed to employ bone densitometry, normally used to identify metabolic bone disorders, to quantify bone mineral density (BMD) from femora belonging to the Coimbra Identified Skeletal Collection. The combination of this technique with a general regression neural network showed good potential for age estimation with an error rate ranging between 9.19 to 13.49 years. This potential was confirmed in a further study^33^ which used clinical data from the National Health and Nutrition Examination Survey. Despite the different populations, the Navega et al.^32^ estimation technique provided a similar accuracy in the result as in the original study^33^. Additionally, computed tomography has been employed to analyse age-related bone loss^34^. Microstructural changes in trabecular bone have shown promising results in a study where age was estimated with R^2^-adj of 0.79 and mean standard error of 6.3 years^35^. Future studies should employ a larger sample size to confirm these results. DNA analysis have also been employed for age estimation with promising results^30,31^. Despite the narrow error (approximately 2 years) these methods remain difficult to apply, time consuming, and heavily affected by contamination and changes in environmental conditions^21^.

Although these are only few examples of age estimation based on bone matrix degeneration, it becomes clear that these are generally reproducible and observer independent. Accuracy standards are still not met in general, yet it is expected that by using a combination of various complementary methods one can achieve a significant improvement in the accuracy of age estimation. The present study explores a multifactorial approach, the potential of which have been previously proposed by Zioupos et al.^36^and Bonicelli et al.^37^. In practice, this study combined nano- and micro-level mechanics including investigation of water, mineral and organic matrix by means of X-ray diffraction, Fourier transformed infrared spectroscopy, and thermal analysis, to provide age at death estimates for a collection of 113 rib cortical bone specimens. The present approach utilises a number of well-known analytical methods, which are combined in an effort to understand age-related changes and has the potential to be used as a multifactorial, observer-independent forensic age estimation method.

## Results

A total of 33 physicochemical parameters were investigated in the present study. Several variables were found to violate normal distribution: optical porosity (PoAr_%_), elastic modulus of osteons (^On^E_IT_), nanohardness of the interstitial bone matrix (^It^H_IT_), enthalpy values for both dehydration and (L∆H) organic combustion (C∆H), mineral to matrix (MM), carbonate substitution (CP), crystallinity index (CI), collagen content (CC) and crystallite Size (Size). This is not expected to affect parametric tests and regression analysis for age estimation due to the large sample size (N > 40).^38^ ANOVA results showed that only two of the variables showed statistical differences between males and females. Crystallite size showed higher value for females (F(1,111) = 1.205 and *p* = 0.040) whereas the enthalpy value for the exothermic peak (C∆H; F(1,111) = 4.746 and *p* = 0.031) was higher for males. The remaining variables showed no significant differences between males and females.

### Correlation between physicochemical and mechanical properties and age

There were also a number of significant correlations between mechanical and physicochemical parameters and age as evaluated by Pearson’s correlation. Scatterplots for visualisation of the relationships are presented in Supplementary Fig. 1–4. The most robust correlation was porosity, which increases linearly with age (R = 0.83 and *p* < 0.001). DSC results (Supplementary Fig. 1) showed that there is a reduction of bone matrix water content associated with increased age, with significant negative correlations for L∆H (R = − 0.27, *p* = 0.004) and W_%_ (R = − 0.25, *p* = 0.007). Furthermore, the enthalpy value for organic matrix combustion (C∆H) was also negatively correlated with age (R = 0.24, *p* = 0.010) and agreed with an age-related decrease in organic content, as shown by weight loss during combustion of organic (Or_%_) matter (R = − 0.39, *p* < 0.001). Finally, mineral content calculated at the very end of the combustion process (Ash_%_) displayed the most robust positive correlation for these TGA variables (R = 0.41, *p* < 0.001). Some features of mineral crystal structure and composition appear to change with increasing age following a linear trend (Supplementary Fig. 2). For instance, carbonate substitution (CP) increases (R = 0.36, *p* < 0.001). XRD analysis shows a general decrease in the lattice size along the ’a’axis (R = − 0.20, *p* = 0.030) and an increase in CL030 (R = 0.23, *p* = 0.016). The segregation of crystallite size and strain by means of Williamson-Hall plot also indicates the increase in crystallite size for the〈00ℓ〉crystallographic direction (R = 0.22, *p* = 0.018). Considering the experimental evidence on bone tissue mechanical behaviour, elastic modulus (E_IT_, R = − 0.19 and *p* = 0.048) and indentation creep (C_IT_, R = − 0.30 and *p* = 0.002) decreases significantly, while indentation work ratio showed a robust negative relationship (η_IT_, R = − 0.41 and *p* < 0.001). Supplementary Fig. 3 shows mean tissue values and Vickers hardness results with age. Similar trends were observed for the osteonal area: ^On^E_IT_ (R = − 0.20 and *p* = 0.032), ^On^C_IT_ (R = − 0.29 and *p* = 0.002) and ^On^η_IT_ (R = 0.40 and *p* < 0.001) show weak correlations with age. For the interstitial area, elastic modulus loses significance while ^It^C_IT_ (R = − 0.27 and *p* = 0.004) and ^It^η_IT_ (R = 0.38 and *p* < 0.001) are still moderately correlated to age. Finally, the two values for microhardness show robust increase with age for osteons (^On^HV, R = 0.44 and *p* < 0.001) and the surrounding interstitial area (^It^HV, R = 0.55 and *p* < 0.001). Thermal analysis parameters also showed clear trends with age. Results for mechanical analysis are visualised in Supplementary Fig. 3, 4.

### Age estimation: unrestricted parameter selection

**Figure 1:**
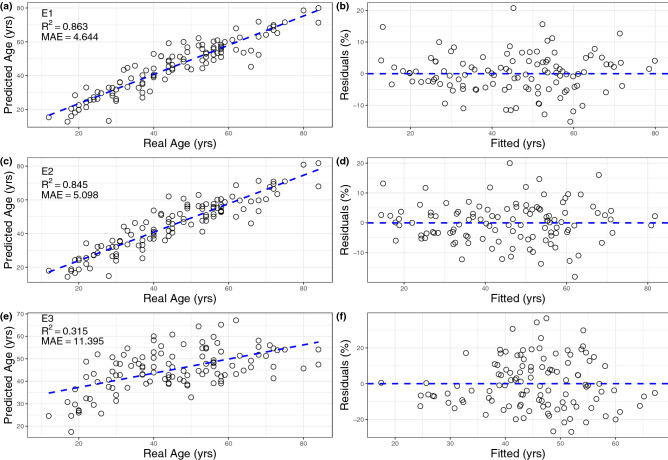
Regression diagnostic plots for the three models. Regression plots presenting the relationship between real age and predicted age for E1 (**a**), E2 (**c**) and E3 (**e**). It is possible to see that there is minimal difference between predicted age for different age ranges. Distribution of residuals is homogeneous for E1 and E2 (**b** and **d**), while E3 does not show regular residuals distribution (**f**).

The aim of this study was to use all available parameters, mimicking a forensic investigation where time and resources are unlimited, with the final goal of reaching maximum accuracy and reliability, as well as meeting the demanding standards required for court admissibility. Stepwise-based parameter selection was employed in order to create optimal regression formulas that produced the best age estimation while maintaining robusticity of the statistical model. In Table 1, E1shows the highest accuracy between all the models with R^2^ of 0.863 and residual standard error (RSE) of 6.453 yrs. Mean absolute error (MAE) is 4.644 years with a maximum residual error of 20.73 for a 49-year-old male individual (Fig. 1a, b). The variables included in this E1 model were selected mainly from nanoindentation, porosity (Po.Ar_%_) and structural parameters crystallite size/geometry obtained from XRD analysis. The only parameter from thermal analysis is C∆H. In terms of experimental procedures, the entire evaluation can be performed in approximately 36 h which would be quick enough to avoid structural and chemical modifications due to storage time and freezing-unfreezing cycles. The only non-significant variable was ’a’axis and none of the parameters violated collinearity restrictions. The model does not violate the assumption for heteroscedasticity (*p* = 0.423). To conclude, residuals were normally distributed (S-W = 0.984, *p* = 0.198) and no autocorrelation was detected (D-W = 1.983, *p* = 0.736).

**Table 1:** Regression coefficient and diagnostic results.

	(E1)	(E2)	(E3)
Po.Ar_%_	6.001***	6.120***	
H_IT_	− 0.446***	− 0.858***	
C_IT_	3.825***		
^On^E_IT_	1.222**	1.683**	
HV		0.531*	
^On^On_IT_	2.523***	1.946**	
^On^HV	0.624***	0.466**	
Ash_%_			4.697***
CΔH	− 0.005***		− 0.012***
CC			204.382***
CL030	5.450***		8.938***
'a'axis	− 369.968		
'c'axis	597.609***		
CL004			− 3.455***
Constant	− 759.525	− 62.45***	− 249.937***
Observations	113	113	113
R^2^	0.863	0.845	0.315
Adjusted R^2^	0.850	0.836	0.283
Residual Std. Error	6.453 (df = 102)	6.737 (df = 106)	14.101 (df = 107)
F Statistic	64.425***(df = 10; 102)	96.441***(df = 6; 106)	9.860***(df = 5; 107)
Akaike Inf. Crit	754.5063	760.5891	926.564
Bayesian Inf. Crit	787.235	782.4082	945.6557

Stepwise regression based on the full set of parameters (E1), only nanoindentation (E2) and physicochemical modifications (E3) (*p < 0.01; **p < 0.05; ***p < 0.001).

### Age estimation: restricted parameter selection

The next step in the analysis is to apply stepwise regression by using two specific subsets (combinations) of parameters in order to address practical problems that can occur in forensic context, such as limitations in time, technical resources, or available bone material. Results for this restricted parameter selection approach can be found in Table 1 with the two formulas E2 and E3. E2 (Table 1) was developed by means of Aikake criterion-based stepwise selection choosing from variables obtained by nanoindentation. The result was an equation (R^2^ = 0.845, R^2^-adj = 0.836) which was produced employing Po.Ar_%_, H_IT_, ^On^E_IT_, HV, ^On^η_IT_ and ^It^HV as independent variables. This approach remains time-consuming (36 h) but reduces the experimentally used instruments to just one. Residual standard error was slightly increased compared to the previous model RSE = 6.737 and mean average error was 5.098 years (Fig. 1c, d). All assumption for residual distribution, heteroskedasticity and autocorrelation were true and valid. Collinearity assumption was not respected for H_IT_ which showed variance inflation factor of 11.24. The final model E3 (Table 1) was built using the automatised stepwise selection based on the entire physicochemical characterisation. In this case, there is a significant reduction in R^2^and an inflation in the RSE = 14.101 and MAE = 11.395 years. The main advantage is the possibility to perform this in only 12 h but accuracy is not sufficient to meet the standard of the forensic settings. Regression plots are shown in Fig. 1e, f.

### Cross-validation

All the models considered the whole cohort of samples and provided, in essence, the maximum possible prediction power of the approach we have implemented. In reality, any unknown sample is not likely to be from the same population which produced the calibration relationship. To simulate this, we applied a leave-one-out method, where in turns one sample was kept out and analysis was produced from the other 113 samples. Results show a minimal decrease in accuracy for all the regression formula with the exception of E3 which had already presented the lowest R^2^ between all the models (Table 2).

**Table 2:** Leave-one-out cross validation results for all the models.

	(E1)	(E2)	(E3)
R^2^	0.863	0.845	0.315
CV—R^2^	0.835	0.825	0.249
RMSE	6.453	6.737	14.101
CV—RMSE	6.736	6.946	14.420
MAE	4.644	5.098	11.395
CV—MAE	5.125	5.428	11.996

It is possible to see a general decrease in all the diagnostic indicators considered to evaluate the regression formulas (CV: cross-validated; RMSE: root mean square error; MAE: mean absolute error).

## Discussion

The indeterminacy in estimating age-at-death of unknown skeletal remains, especially from mature individuals, is ascribable to the high biological variability associated with the maturation/degeneration process^39^. The traditional age estimation methods based on skeletal morphology, development or degeneration are often subjective or heavily biased and do not fulfil the newly established standards for expert witness admissibility. A number of approaches based on chemical and molecular methods have been developed in order to overcome these limitations. The present study evaluated the effectiveness of cortical bone matrix analysis for age estimation. This method employs a multifactorial approach starting from a pool of physicochemical parameters related to water, collagen and mineral acquired by physical and chemical methods and indentation mechanical parameters. However, not all the parameters showed sufficient correlations with age in order to predict age at death and therefore stepwise regression was applied in order to produce optimal estimation formulae. Results are promising and, although the lowest MAE is 4.644 years, there is no noticeable decrease in accuracy for individuals after maturity (Table 2). Equations E1 (based on the entire set of parameters, Fig. 1a, b), and E2 (obtained from histomorphometry and mechanical analysis, Fig. 1c, d), were deemed the most suitable for age estimation. Estimates based only on chemical analysis did not provide the necessary accuracy showing age estimate deviations of > 10 years (Fig. 1e, f).

This discrepancy between chronological and tissue age of the bone matrix has been attributed to alterations in modelling/remodelling rate due to physiological maturing and pathological conditions (e.g., osteoporosis). Age-related changes in one of the matrix components (mineral, organic or water) has a profound effect on the others and in the mechanical behaviour of bone from the nano- to macro-scale. The increase in mineral content is the most apparent change. However, this is related to a number of modifications in crystallite size and overall crystallinity^40–43^. This is in part due to the effect of impaired remodelling with age, osteocalcin^43,44^, and changes in hydroxyapatite composition, such as the reduction in a-axis length with the increase in carbonate substitution as well as the increase in overall crystal size^41,45–47^. In the present study, there is a robust increase in mineral content (R^2^ = 0.41 and *p* < 0.001) accompanied by the increase in carbonate substitution, a trend which has recently been confirmed by Pedrosa et al.^29^ Results for the present study support previous findings showing that mineralisation increases until maturity is reached. After this point, approximately 35 years, the variation in mineral content remains stable for the rest of the age range^48–50^. These results are supported by the decrease in both water and collagen content. These variations in matrix composition result in changes in the functional mechanical behaviour. Despite the fact that weak or no correlation has been previously found between nanohardness and elastic modulus^51^, the present study reveals a significant decrease in the elastic modulus of bone with the exception of interstitial bone^52^. Furthermore, this is coupled with the decrease in crystal strain values and an increase in elastic indentation work ratio with age. Finally, microhardness showed a significant increase with age for both osteon and interstitial bone and was strongly associated with increase in mineral content. This suggests that microstructure (e.g., number, thickness and mineralisation of lamellae) along with age, could be a key factor in determining micromechanical properties.

In terms of age estimation, E1 (in Fig. 1a, b) shows the best prediction between all models produced along the entire age range. Age-at-death estimates from this model, however, require the longest time to produce (approximately 36 h) and involves a large number of analytical tests, both physicochemical and mechanical, which may not be always easily accessible. Regardless this model (E1) stands out as one of the best methods for age estimation. With a mean absolute error of 4.64 years, it is comparable to aspartic acid racemisation.

Other comparable and commonly used age estimation methods include Griffin et al.^53^, who developed a procedure based on teeth with the potential accuracy of ± 8.7 years for all ages and ± 6.2 for under 35 individuals. Alkass et al.^54^ achieved ± 1 year of deviation from real age by radiocarbon and ± 5.4 for aspartic acid racemization. However, one must consider the fact that the first methodology was developed for a collection of just 39 teeth^53^ while the second on 66 tooth samples^54^ which are hardly representative of a larger population and might retain a certain degree of uncertainty, while the present study is based on almost double the number of specimens allowing for a more reliable estimation. Recently researchers have utilised DNA analysis increasingly due to its high accuracy despite the destructive nature of the procedure^21,22,55^. Xu et al.^30^ tested 2,957 novel age-associated DNA methylation sites and claimed that a certain combination of 11 sites could be used to estimate age with an absolute error of ± 2.8 years. These methodologies, despite the high effectiveness, require specific training and facilities to be carried out and are more sensitive to environmental conditions and external contamination than the present one.

In order to limit the complexity of the experimental procedures and reduce execution time E2 (Fig. 1c, d) was developed involving only histomorphometry and mechanical analysis. Loss in accuracy is minimal, as can be seen in Table 2, and the entire experimental procedure can be carried out in less than 24 h. Though the present method has not yet been tested extensively for the possible bias of diagenesis, it was found that, for short *postmortem* intervals, it has only a marginal effect on micro- and nano-mechanical matrix properties^56^. Additionally, the overall physicochemical analysis based on powder only in isolation does not provide the necessary accuracy for the forensic setting (E3 in Table 1), making E2 the preferable option for a model that combines both speed of execution and desirable results. Finally, as expected, leave-one-out cross-validation (Table 2) for all of the models was overall only slightly less accurate, suggesting that this approach produces robust and reproducible estimations.

Compared to the two previous pilot studies based on the same approach, as shown in Table 3^36,37^_,_ the most evident difference is the higher number of specimens involved: 113 for the present study compared to the 24 in Bonicelli et al.^28^ and 14 in Zioupos et al*.*^27^. This approximately tenfold-increase in sample size is essential in order to confer robusticity to the regression models based on a multifactorial approach^57^. An expected effect of this difference is the decrease in accuracy (from R^2^ = 0.99 to R^2^ = 0.86) as well as the increase in standard error of the estimation. This can be attributed to the broader age range of this study (12 to 84) as compared to Bonicelli et al.^28^ (20–68 years) and Zioupos et al.^27^ which only involved mature individuals (> 35 years of age). Additionally, this study’s methodology is accurate across the entire age range, a clear advantage for applicability on unknown skeletal remains.

**Table 3:** Commonly used age-at-death methodologies.

Reference	Method	R^2^	RE	SDE
E1	Rib (n = 113)	0.863	4.64	-
Bonicelli et al*.*^37^	Rib (n = 24)	0.949	2.14	0.4
Zioupos et al*.*^36^	Femur (n = 14)	0.997	0.6	0.31
Griffin et al*.*^53^	Teeth (n = 31)	0.92	8.7	-
Alkass et al*.*^54^	Teeth (n = 57)	0.99	1.88	1.3
Huang et al*.*^55^	Blood (n = 89)	0.819	7.87	-
Bekaert et al*.*^31^	Blood (n = 206)	0.95	3.75	-
Bekaert et al*.* ^31^	Teeth (n = 29)	0.74	4.86	-
Martin-De Las Heras et al*.*^28^	Teeth (n = 22)	0.65	14.9	-

Comparison of accuracy and error of the estimation of the main laboratory-based approaches in age estimation (R^2^: coefficient of determination; RE: mean absolute residual error; SDE: standard deviation of absolute residual errors).

The present approach is not without limitations. The variation of mechanical behaviour of rib cortical bone has been investigated by Agnew et al.^58,59^, in a study that simulated frontal impact on 70 rib specimens. They reported that age does not explain the majority of the variance but other factors at individual (e.g., sex, BMI) and structural (e.g., geometry and microstructure) levels need to be considered during ageing. Furthermore, it has been proved that there is high regional variation due to the local geometrical properties of the area tested^60,61^. This would suggest that macroscopic mechanical properties may not be suitable for age estimation. However, the method developed in the present study employs only indentation testing, which has a lower intra-site variation as compared to dynamic macroscopic testing^62^. Further, diagenesis could have an impact on all analytical parameters produced in the various methods and tests employed on fresh bone specimens. The same can be said, however, for all alternative methodologies such as aspartic acid racemisation or DNA. Biological variability and medical history are unavoidable complications. Concerning chemical analysis, infrared spectroscopy has been used in the past to evaluate bone diagenesis^63,64^, revealing that there are changes in both the organic phase (reduction in collagen and protein content) and the mineral phase (crystallite composition, size and crystallinity). This could in turn have a systematic effect on thermal analysis and XRD. It is therefore essential that further studies focus on the influence of diagenesis on the model estimates and the ideal conditions in which the method should be applied. Diagenetic changes have also been shown to influence bone mechanical behaviour, but a specific test applied to this methodology to evaluate the impact of taphonomy on the present protocol should be developed. It is worth mentioning that we found, although on a smaller sample size, no difference between Albanian and Greek samples in a previously published study^37^. This suggests that the approach is not heavily affected by population differences. However, it has been proven that the variables under analysis are highly dependent on the remodelling rate and thus they may be connected with genetic factors linked to population differences. Moreover, environmental factors such as diet and activity could play a crucial role in affecting bone matrix behaviour, thus, further research is necessary to investigate all these sources of bias in the estimates. Lastly, male to female numbers in this study were not balanced, a fact that could have caused some unknown bias and may require further consideration. Despite the aforementioned limitations the current study represents the first and largest study to consider five different methods of assessing changes in bone matrix as a function of age. This attempt resulted in a reliable, objective and accurate age estimation method with great potential to become widely applicable in forensic settings. Naturally, follow-up validation studies are needed to confirm the present results to make it applicable for forensic applications.

To conclude, the present study introduces the first comprehensive multifactorial approach based on physicochemical and mechanical modifications of bone matrix. Its main advantages are it is observer independent and easy to apply, even by an operator who does not possess forensic expertise. Furthermore, the protocol only requires a small bone volume and it could be carried out by shipping the unknown sample to a fully equipped laboratory. Despite being a study based exclusively on fragments of one skeletal element, the method showed satisfactory accuracy and therefore it meets the three main requirements for expert witness admissibility in forensic settings. The approach, developed on fresh samples, needs further testing in order to understand the effect of post-mortem intervals, any effects of taphonomy and the potential bias introduced by pathological conditions (e.g., osteoporosis, osteogenesis imperfecta). Finally, a larger number of reference bones should be targeted in order to optimise applicability of the approach.

## Materials and methods

The skeletal material employed in this study consists of 113 sternal ends of the fourth right rib, approximately 5 cm in length, with known ages ranging from 12 to 84 years (46.64 ± 16.33 years), all collected at the Institute of Forensic Medicine in Tirana, Albania. Seventy-seven male individuals (45.23 ± 16.66 years) and 36 females (44.57 ± 16.88 years) were used in total (see Supplementary Table 1).

### Bone specimen preparation

The autopsy material, sampled for the purpose of the present study at the Institute of Forensic Medicine of Tirana after permission was granted by the Ministry of Justice of Albania, was received in dry ice and kept at − 20 °C between extraction, preparation and experimental procedure. The samples were allowed to dry completely in order to avoid structural modifications due to the increase in volume of water when freezing. The experimental procedure was carried out ensuring minimal storage time and avoiding repeated freezing and defrosting cycles. A Struers Accutom wafering saw equipped with a diamond impregnated blade (300 µm) cooled down using deionised water was used to produce two, 3 mm thick, cross sections of the sternal portion of the rib. The sections were high pressure washed to remove bone marrow and then degreased using a solution of chloroform–methanol in the ratio 1:1 for 36 h. Subsequently, the samples were immersed in 100% ethanol for 12 h and left overnight to dry at room temperature. After drying for 24 h at room temperature, the sections were embedded in epoxy resin (MetPrep Kleer Set Type SSS) and metallographically polished using an automatic Struers RotoPol-15 with 203 mm silicon carbide abrasive disks grinding paper of decreasing grit size (400, 800, 1200, 2500) on a MasterTex cloth with Alumina 3B 6 oz. The result is a mirror-like surface that enables magnification × 20.

The remaining material was divided in two parts using the wafer edge saw. Trabecular bone and periosteum were scraped off using a scalpel. The bone was treated following the chloroform–methanol procedure explained in the above paragraph. In order to obtain the powder, the material was processed using a Retsch Mixer mill 2000 by cycling for 1 min and then at 60 Hz. In between the two different cycles, the powder was filtered using a 106 µm sieve to guarantee particle homogeneity. The powder was left resting at room temperature overnight before testing. In order to maintain consistency throughout the study, the same particle size (106 µm) was used for chemical characterisation.

### Mechanical characterisation and histomorphometry

**Figure 2:**
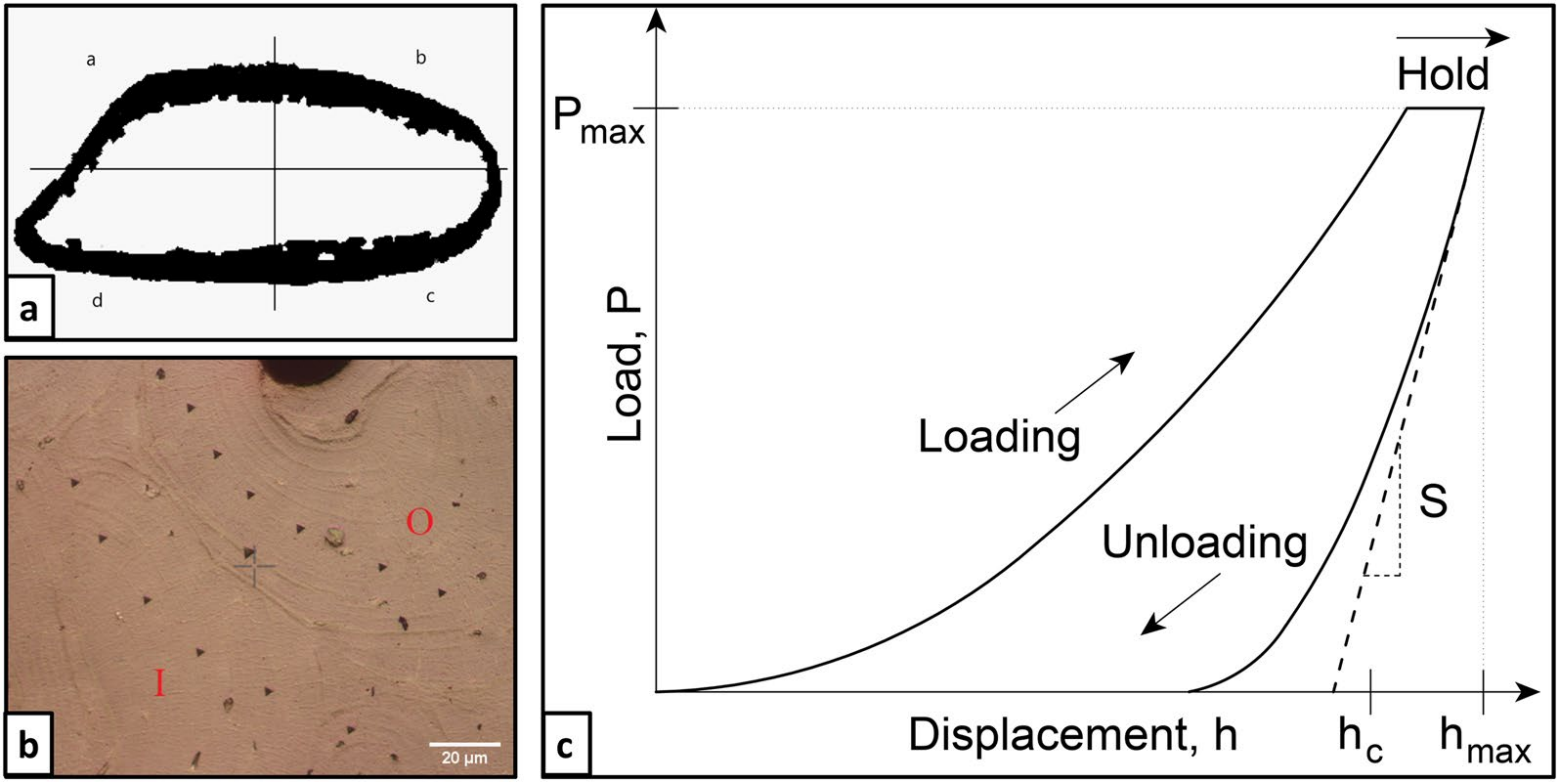
Steps for nanoindentation. The four quadrants used to divide the cortical area for sampling in order to perform porosity calculation (**A**) and nanoindentation impressions (**B**) on the osteon (O) and the surrounding interstitial bone (I) divided by the cement line (red dashed line). (**C**) Shows the typical load/indentation depth curve used to acquire mechanical parameters.

Nanoindentation was performed using a CSM-NHT (system v.3.75, CSM, 2034 Peseux, Switzerland) instrument. Maximum hold load was set at 10 mN with a loading and unloading speed of 20 mN/min with 30 s long load/hold/unload phased experiment. The cortical area was divided in four quadrants (two on the cutaneous and two on the pleural surface). For each quadrant, eight indentations were placed on one osteon (On) and on the surrounding interstitial matrix (It). The three steps of nanoindentation testing, from location targeting to curve acquisition, can be seen in Fig. 2A–C. The target sites were chosen according to the regularity of the surface. Mean tissue values were also calculated based on the average between the mechanical properties of the two areas. Universal hardness (H_IT_ in Vickers) was calculated from load and contact area in Eq. (1).

1 $${H_{IT}} = \frac{{{P_{max}}}}{A}$$

where P_max_ is maximum load and A is the total area of the impression resulting from the indentation. Elastic modulus (E_IT_, GPa) was obtained (assuming Poisson's ratio value of ν = 0.3) in the unloading phase as per the Oliver and Pharr method, Eq. (2).

2 $${E}_{IT}={\left[\frac{1- {\upnu }_{s}^{2}}{{E}_{s}}+ \frac{1- {\upnu }_{i}^{2}}{{E}_{i}}\right]}^{-1}$$

where ν_i_ and E_i_ refer to the Poisson's ratio and the elastic modulus of the indenter respectively, and ν_s_ to the Poisson's ratio in the sample, assuming that the latter is homogeneous. Indentation creep C_IT_ was calculated by the proportional increase in depth occurring while the load is held at its maximum level (for 30 s) and its measurement reflects the visco-plasticity of the tissue shown in Eq. (3).

3 $${C}_{IT}= \frac{{h}_{1} - {h}_{2}}{{h}_{1}} \times 100$$

The elastic portion of the indentation work η_IT_ was obtained by examining the percentage ratio of the elastically recovered energy over the total (elastic + plastic) energy input during an indentation sequence, Eq. (4).

4 $${\eta }_{IT}=\frac{{w}_{elast}}{{w}_{elast}+{w}_{plast}}\times 100$$

An INDENTEC HWDM-7 instrument, equipped with a square-shaped pyramid diamond tip of θ = 136°, was employed to produce Vickers microhardness (HV, Kg/mm^2^) values for osteonal and interstitial bone areas for each specimen. The maximum load in these tests was set at 10 gf. The same areas selected for nanohardness testing were examined applying one indentation on the osteon and one on the surrounding matrix for a total of eight indentations per section.

Optical porosity (Po.Ar, %) was obtained from four images taken for each specimen with a reflected light microscope × 20 and the use of ImageJ^65,66^. Four locations were selected in order to sample the entire cortical area in the four quadrants, two sites on the pleural surface and two from the cutaneous surface. Each image was cropped to select areas completely occupied by bone and converted into 16-bit images. A threshold mask was applied to highlight the areas not occupied by bone tissue. The volume fraction was calculated using the open source software BoneJ^65,66^ and was then transformed into a percentage value. The values obtained for the four locations tested in each individual were averaged in order to obtain a mean measurement for optical porosity. Osteocyte lacunae were included when automatically selected by the software. When thickness of cortical area did not allow accurate measurement of porosity due to excessive bone resorption the measurement was taken manually. With the segmented line tool, total area was calculated. The surface occupied by vascular canals was measured in the same manner. Bone area was obtained subtracting vascular areas from total cortical area. Porosity was assessed as a percentage by dividing bone area by total cortical surface.

### Thermal analysis

**Figure 3:**
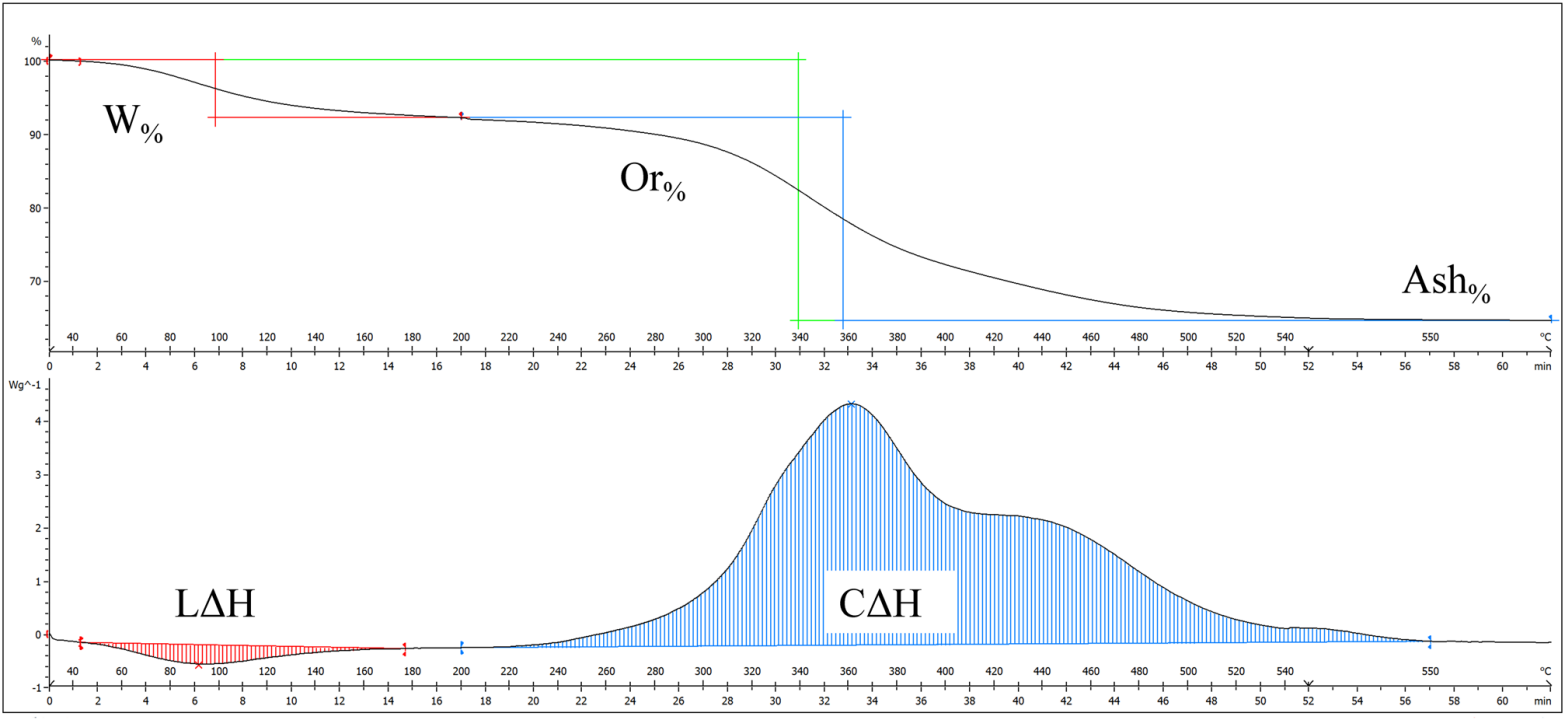
Graphic representation of thermal analysis experiment. Example of thermogravimetric (**A**) and differential scanning calorimetry (**B**) curves. The first weight loss represents the bone matrix dehydration and matches with the endothermic peak responsible for triple helix thermal denaturation (LΔH and W%). The second weight loss (Or%) represents organic combustion (CΔH) and matches the DSC curve. Subtracting the entire weight loss (Ash%) from 100, it is possible to obtain mineral content.

Thermal analysis was carried out by using a TGA/DSC 3 + (Mettler Toledo , Indium calibrated) with a two-phase experiment: (i) dynamic temperature increase from 25 to 550 °C at a rate of 10 °C/min and (ii) a static phase in which 550 °C was sustained for 10 min in order to completely eliminate the organic matrix and reduce the bone to ashes (Fig. 3). Temperature in the chamber was controlled by a continuous flow of water at room temperature. The powder was tested in air in 40 µL aluminium pans with flat bases filled with approximately 10 mg of bone powder and the weight was recorded using a microbalance (Sartorius Genius ME235), while an empty crucible was used as a reference. STAR^e^ version 16.00 was used for the curve analysis. Thermogravimetric curves (TGA) were divided in three temperature ranges and step horizontal was used to calculate percentage weight loss. Dehydration of the sample (W_%_, %) was calculated between ~ 25 and 200 °C and organic weight loss (Or_%_, %) between ~ 200 and 550 °C. The sum of the two steps represents the entire weight loss and subtracting it from 100 gives the mineral content percentage (Ash_%_, %). The same intervals were used to calculate enthalpy values from differential scanning calorimeter curves by means of linear integration. The first endothermic episode represents the energy required to dehydrate the bone and break hydrogen bonds (LΔH, Wg^−1^) while the second exothermic episode represents organic combustion (CΔH, Wg^−1^). These intervals were chosen according to literature^36,37,67^ and are shown in Fig. 3.

### Fourier transform infrared spectroscopy

In this study, spectra were collected by means of ALPHA T Platinum spectrometer (Bruker Optics) in attenuated total reflectance mode (ATR). The range analysed is 4000–400 cm^−1^ with 4 cm^−1^ resolution for a total of 64 scans. Approximately 3 mg (~ 106 µm particle size) of bone powder was analysed. The stage and crystal were cleaned with deionised water before the first and after each measurement. The measurements for the entire sample were carried out in the same conditions to maximise consistency. Spectral analysis was performed in the open source software SpectraGryph version 1.2.15. The baseline was calculated for each peak individually and the areas under the peak and intensity values were obtained. The variables considered in this study were the mineral to matrix ratio, the carbonate to phosphate ratio, the crystallinity index, and the collagen content. Calculations for all these variables are shown in Table 4.

**Table 4:** ATR-FTIR variables.

Parameter	Abbreviation	Explanation
MM	A_1200-900_/A_1750-1600_	Integrated values of v1v3 phosphate over Amide I band^70^
CP	A_890-850_/A_1200-900_	Integrated values of v2 carbonate over v1v3 phosphate bands^70^
CI	I_605_ + I_565_/I_595_	Mineral crystallinity index calculated on the v4 phosphate peak^71^
CC	A_1750-1600_/A_1200-900_	Integrated values of Amide I band v1v3 phosphate band^72^

Description of the semiquantitative analysis of FTIR spectra (A: area of the peak; I: intensity of the peak: MM: mineral to matrix ratio; CP: carbonate to phosphate ratio; CI: crystallinity index; CC: collagen content).

### X-ray diffraction

This study employed a pXRD sample holder with a glass spacer filled with bone powder for XRD analysis and a PANalytical X’Pert Pro Multi-Purpose Diffractometer by means of Cu Kα radiation source for the characterisation. Data collection was carried out across an angular range of 10–80 2θ(°) (8.84–1.20 d-spacing) using a PIXcel strip detector at count rate ∼1 s. Data were also collected for two further stepped scans under the same sample conditions but across an angular range of 23–27 2θ(◦) (3.86–3.30 d-spacing) and 50–55 2θ(◦) (1.82–1.67 d-spacing), and with a count time at each step equivalent to ∼3 s. The two additional stepped scans were collected to provide greater quality data at the 002 and 004 Bragg maxima respectively. Asymmetrical split pseudo-Voigt (SPV) peaks were fit to the 002, 004, 030 and 210 diffraction maxima (as it represents a satisfying compromise between intensity, overlapping reflections and differing lattice direction) for all diffraction data. SPV peaks approximate a Voigt function which is a combination of Gaussian and Lorentzian peaks. From this data, the full width at half maximum (FWHM) of the 002 and 004 Bragg maxima was calculated. FWHM was also calculated from the 10–80 angular range for the 030 and 210 peaks. The FWHM values were to calculate coherence length using the Scherrer equation. In order to control the instrument resolution factor, a silicon standard (NB1640) was measured weekly and the factor was calculated by means of Caglioti equation. Bruker Topas software (Version 4.1, 2008) was used for fitting each diffraction profile. This provided quantitative crystallite size and morphology parameters through calculation of the coherence length and structural parameters of the crystal lattice. Coherence length was calculated for three orthogonal crystallographic directions:〈00ℓ〉,〈hk0〉and〈0k0〉using the Scherrer equation, which uses the instrument corrected FWHM of the desired peak. After fitting the peaks, FHWM was calculated and used to obtain coherence length (CL in Eq. (5)) at all crystallographic directions under investigation.

5 $$CL= \frac{K\lambda }{\beta \mathrm{cos}\theta }$$

where K is the Scherrer constant (0.9), λ is the X-ray wavelength (0.15406 nm) and θ is the Bragg angle. Coherence length is a calculation of both the size and strain of the crystal, showing positive relationship with size and negative with strain. The lattice parameters were calculated from whole pattern fitting refinement of diffraction profiles to obtain the 2θ peak positions. Considering the difficulties in analysing peak broadening for biogenic hydroxyapatite, coherence length for〈00ℓ〉crystallographic direction was separated in crystallite size and microstrain using the 002 and 004 maxima by means of the indirect Williamson-Hall plot according to Eq. (6), where βis the FWHM, L is the crystallite size, and ε is the microstrain^68,69^.

6 $$\beta \mathrm{cos}\theta =4\varepsilon \mathrm{sin}\theta +\frac{k\lambda }{L}$$

### Statistical analysis

Statistical analysis was carried out in R 3.6.0. First, normality was investigated using Shapiro–Wilk Test (S-W) with significance value set at ≤ 0.05. Analysis of variance (ANOVA) were also applied to evaluate mean differences considered significant for p ≤ 0.05. A preliminary inspection of the correlation between age and the entire set of predictors was carried out with Pearson's correlation (≤ 0.05). Stepwise AIC regression (using https://github.com/rsquaredacademy/olsrr) was then applied to the entire set of parameters (unrestricted parameter selection) which built regression models from a set of candidate predictor variables by entering and removing predictors based on Akaike Information Criteria. This aims to achieve the maximum accuracy without considering time and facilities limitation. The same procedure was applied separately for nanoindentation and full physicochemical characterisation (thermal analysis, FTIR and XRD) data, in order to simulate forensic cases where there may be limited time and resources available for analysis. All the models were checked for collinearity, variance inflation factor, condition index and heteroscedasticity (Breusch-Pagan test Bonferroni adjusted). Residuals diagnostics were carried out visually by means of residual QQ plots, Residual vs Fitted Values plots, Scale-Location plots, Shapiro–Wilk normality tests (S-W) and Durbin-Watson tests for residual autocorrelation (D-W). Finally, outliers were detected by Cook's Distance bar plot to evaluate the effect of different antemortem conditions on the age-at-death estimation. To conclude, leave-one-out (LOO) cross-validation was applied to all regression models to avoid splitting the sample (https://github.com/topepo/caret). Cross-validated results were compared to the initial regression results by means of R^2^, root mean square error and mean absolute error. This way it was possible to evaluate the concentration of residuals along with the best fit after cross-validation. The entire set of variables and descriptive statistic can be found as Supplementary Table 2.

### Ethical approval

Permission for sampling was granted to the Institute of Forensic Medicine in Tirana (Protocol Number 795/3 A. Xh.) and the Ministry of Justice in Albania for the current study. Albania’s local regulations had no condition for informed consent in minimal sampling that was required for the study as long as anonymisation of the samples was guaranteed. This condition was met. The study protocol was approved by the Ethics Committee of the University of Edinburgh. All methods were carried out in accordance with the approved guidelines and the appropriate standards applying in the medico-legal context. The material was further cleared by the NHS Lothian Tissue Governance (reference number ICA01/17). Additionally, ethical approval was provided by Cranfield University Ethics Committee (CURES/2294/2017) to carry out experimental procedures.

## Supplementary Information

Supplementary files.

## Supplementary Information

The online version contains supplementary material available at 10.1038/s41598-021-81342-0. Data underlying this paper can be accessed at CORD (Cranfield Online Research Data) 10.17862/cranfield.rd.13516532.v1.
