# Supplementary material for: Age related changes of rib cortical bone matrix and the application to forensic age-at-death estimation
Source: Sci Rep. 2021 Jan 22;11:2086. doi: 10.1038/s41598-021-81342-0 (PMC7822937; doi:10.1038/s41598-021-81342-0)
Supplement: Supplementary file 1 — Supplementary files. [file 41598_2021_81342_MOESM1_ESM.docx]

**Age Related Changes of Rib Cortical Bone Matrix and the Application to Forensic Age-at-Death Estimation**

Andrea Bonicelli^1,2^, Peter Zioupos^2^, Emily Arnold^3^, Keith D. Rogers^3^, Bledar Xhemali^4^, Elena F. Kranioti^5^

*^1^Edinburgh Unit for Forensic Anthropology, School of History Classics and Archaeology, University of Edinburgh, Edinburgh, United Kingdom*

*^2^Musculoskeletal & Medicolegal Research Group, Cranfield Forensic Institute, Defence Academy of the UK, Shrivenham, United Kingdom*

*^3^Materials Science and Radiation Group, Cranfield Forensic Institute, Defence Academy of the UK, Shrivenham, United Kingdom*

*^4^Institute of Forensic Medicine, Tirana, Albania*

*^5^Department of Forensic Sciences, Faculty of Medicine, University of Crete, Heraklion, Greece*

| **Code** | **Cause of Death** | **Sex** | **Age** |
| --- | --- | --- | --- |
| Rib1 | Gunshot wound | Male | 33 |
| Rib2 | Sudden death | Female | 49 |
| Rib3 | Traffic accident | Male | 19 |
| Rib4 | Sudden death-cardiac infarction | Male | 49 |
| Rib5 | Craniocerebral trauma-traffic accident | Male | 24 |
| Rib6 | Gunshot wound | Female | 29 |
| Rib7 | Occupational accident | Male | 24 |
| Rib8 | traffic accident-car | Male | 45 |
| Rib9 | Hit by lightening | Female | 28 |
| Rib10 | self-poisoning | Female | 20 |
| Rib11 | Fall from height | Female | 43 |
| Rib12 | Traffic accident-car | Male | 44 |
| Rib13 | self-poisoning | Female | 37 |
| Rib14 | Hanging | Male | 58 |
| Rib15 | Sudden death | Male | 23 |
| Rib16 | Calcium oxide death (quicklime) | Male | 44 |
| Rib17 | Traffic accident-car | Male | 25 |
| Rib18 | Hanging | Female | 31 |
| Rib19 | Fall from height | Female | 40 |
| Rib20 | Self-poisoning | Female | 37 |
| Rib21 | Traffic accident-car | Male | 12 |
| Rib22 | Gunshot wound | Male | 44 |
| Rib23 | Self-poisoning | Male | 56 |
| Rib24 | Self-poisoning | Male | 30 |
| Rib25 | Traffic accident-car | Male | 33 |
| Rib26 | Gunshot wound | Male | 37 |
| Rib27 | Gunshot wound | Male | 20 |
| Rib28 | Burned | Female | 44 |
| Rib29 | Traffic accident-car | Male | 59 |
| Rib30 | Suicide by poisoning | Female | 19 |
| Rib31 | Sudden death | Male | 58 |
| Rib32 | Sudden death | Male | 50 |
| Rib33 | Sudden death | Male | 53 |
| Rib34 | Traffic accident-car | Male | 34 |
| Rib35 | Traffic accident-car | Male | 36 |
| Rib36 | Fall from height | Female | 37 |
| Rib37 | Sudden death | Female | 56 |
| Rib38 | Traffic accident-car | Male | 54 |
| Rib39 | Self-poisoning | Male | 26 |
| Rib40 | Falling from height | Male | 18 |
| Rib41 | Drowning | Male | 17 |
| Rib42 | Traffic accident-car | Male | 18 |
| Rib43 | Drowning | Female | 31 |
| Rib44 | Traffic accident-car | Male | 45 |
| Rib45 | Sudden death | Male | 53 |
| Rib46 | Traffic accident-car | Male | 30 |
| Rib47 | Self-poisoning | Male | 58 |
| Rib48 | Cerebral contusion | Male | 49 |
| Rib49 | Acute myocardial infraction | Female | 66 |
| Rib50 | Overdose | Male | 40 |
| Rib51 | Cerebral trauma, car accident | Male | 72 |
| Rib52 | Self-poisoning | Female | 42 |
| Rib53 | Cerebral haemorrhage | Male | 71 |
| Rib54 | Cerebral contusion | Male | 37 |
| Rib55 | Acute myocardial infraction | Male | 48 |
| Rib56 | Acute myocardial infraction | Male | 62 |
| Rib57 | Infectious disease | Male | 25 |
| Rib58 | Asphyxia | Male | 35 |
| Rib59 | Acute myocardial infraction | Male | 73 |
| Rib60 | Acute myocardial infraction | Female | 66 |
| Rib61 | Vertebral trauma | Male | 68 |
| Rib62 | Multiorgan disfunction | Female | 56 |
| Rib63 | Self-poisoning | Male | 75 |
| Rib64 | Haemorrhagic shock | Female | 84 |
| Rib65 | Acute myocardial infraction | Female | 72 |
| Rib66 | Self-poisoning | Female | 58 |
| Rib67 | Asphyxia | Female | 53 |
| Rib68 | Cerebral trauma, car accident | Male | 22 |
| Rib69 | Acute myocardial infraction | Male | 72 |
| Rib70 | Haemorrhagic shock, fall | Male | 29 |
| Rib71 | Self-poisoning | Male | 63 |
| Rib72 | Cerebral trauma, fall | Female | 52 |
| Rib73 | Cerebral trauma, car accident | Male | 22 |
| Rib74 | Asphyxia | Female | 40 |
| Rib75 | Acute myocardial infraction | Male | 54 |
| Rib76 | Haemorrhagic shock, fall | Male | 56 |
| Rib77 | Acute myocardial infraction | Female | 80 |
| Rib78 | Haemorrhagic shock, car accident | Female | 68 |
| Rib79 | Self-poisoning | Male | 57 |
| Rib80 | Self-poisoning | Female | 42 |
| Rib81 | Cerebral haemorrhage, car accident | Male | 70 |
| Rib82 | Stabbing | Male | 54 |
| Rib83 | Acute myocardial infraction | Female | 61 |
| Rib84 | Overdose | Male | 64 |
| Rib85 | Acute myocardial infraction | Male | 32 |
| Rib86 | Acute myocardial infraction | Male | 56 |
| Rib87 | Acute myocardial infraction | Male | 45 |
| Rib88 | Self-poisoning | Female | 40 |
| Rib89 | Ventricular rupture | Male | 53 |
| Rib90 | Sudden death | Female | 56 |
| Rib91 | Haemorrhagic Shock, Fall | Male | 84 |
| Rib92 | Sudden death | Female | 39 |
| Rib93 | Traffic accident | Male | 38 |
| Rib94 | Asphyxia | Male | 44 |
| Rib95 | Gunshot wound | Male | 30 |
| Rib96 | Mechanical asphyxia | Male | 30 |
| Rib97 | Gunshot wound | Female | 29 |
| Rib98 | Sudden death | Male | 57 |
| Rib99 | Traffic accident | Female | 58 |
| Rib100 | Asphyxia | Male | 48 |
| Rib101 | Self-poisoning | Male | 45 |
| Rib102 | Sudden death | Female | 54 |
| Rib103 | Sudden death | Male | 58 |
| Rib104 | Gunshot wound | Male | 68 |
| Rib105 | Traffic accident | Male | 28 |
| Rib106 | Traffic accident | Male | 40 |
| Rib107 | Traffic accident | Female | 22 |
| Rib108 | Traffic accident | Female | 40 |
| Rib109 | Asphyxia | Male | 20 |
| Rib110 | Sudden death | Male | 62 |
| Rib111 | Asphyxia | Male | 47 |
| Rib112 | Acute myocardial infraction | Male | 48 |
| Rib113 | Sudden death | Male | 52 |

**Supplementary Table 1.** Demographic for the entire sample involved in the study including sex, age and cause of death.

| Parameter | Abbreviation | Instrument | Unit | Mean | Median | SD |
| --- | --- | --- | --- | --- | --- | --- |
| Optical porosity | Po.Ar% | ImgeJ | % | 5.95 | 5.85 | 0.2 |
| Indentation nanohardness | H | Nanoindentation | Vickers | 48.11 | 48.11 | 0.87 |
| Nanoindentation modulus (for Poisson’s ratio ν = 0.3) | E | Nanoindentation | GPa | 18.96 | 19 | 0.2 |
| % indentation creep at hold (contact load) | C_IT_ | Nanoindentation | % | 5.13 | 5.2 | 0.07 |
| Elastic work % over the total (elastic + plastic) indentation energy | η_IT_ | Nanoindentation | % | 21.27 | 21.14 | 0.17 |
| Indentation nanohardness for osteons | ^On^H | Nanoindentation | Vickers | 46.01 | 45.29 | 0.88 |
| Nanoindentation modulus (for Poisson’s ratio ν = 0.3) for osteons | ^On^E | Nanoindentation | GPa | 17.75 | 17.75 | 0.2 |
| % indentation creep at hold (contact load) for osteons | ^On^C_IT_ | Nanoindentation | % | 4.93 | 4.93 | 0.07 |
| Elastic work % over the total (elastic + plastic) indentation energy for osteons | ^On^η_IT_ | Nanoindentation | % | 21.69 | 21.57 | 19.38 |
| Indentation nanohardness for interstitial bone | ^It^H | Nanoindentation | Vickers | 50.22 | 49.38 | 0.94 |
| Nanoindentation modulus (for Poisson’s ratio ν = 0.3) for interstitial bone | ^It^E | Nanoindentation | GPa | 20.17 | 20.03 | 0.23 |
| % indentation creep at hold (contact load) for interstitial bone | ^It^C_IT_ | Nanoindentation | % | 5.33 | 5.37 | 0.06 |
| Elastic work % over the total (elastic + plastic) indentation energy for interstitial bone | ^It^η_IT_ | Nanoindentation | % | 20.84 | 20.81 | 0.17 |
| Microhardness for osteons | ^On^HV | Microindentation | Kg/mm^2^ | 33.36 | 33.75 | 0.48 |
| Microhardness for interstitial bone | ^It^HV | Microindentation | Kg/mm^2^ | 39.2 | 39.43 | 0.47 |
| Microhardness | HV | Microindentation | Kg/mm^2^ | 41.79 | 41.9 | 0.49 |
| Enthalpy measurement of the endothermic episode | LΔH | TGA/DSC3+ | Wg-1 | 159.58 | 156.93 | 1.7 |
| Enthalpy measurement of the exothermic episode | CΔH | TGA/DSC3+ | Wg-1 | 3309.61 | 3276.54 | 26.76 |
| Water weight loss | W% | TGA/DSC3+ | % | 8.63 | 8.59 | 0.07 |
| Organic weight loss | Or% | TGA/DSC3+ | % | 28.1 | 28.02 | 0.1 |
| Value of the final weight | Ash% | TGA/DSC3+ | % | 63.27 | 63.51 | 0.14 |
| Mineral to matrix ratio | MM | ATR-FTIR | ratio | 6.82 | 6.97 | 0.7 |
| Carbon substitution | CP | ATR-FTIR | ratio | 0.019 | 0.019 | 0.002 |
| Crystallinity index | CI | ATR-FTIR | ratio | 0.99 | 1.01 | 0.006 |
| Collagen content | CC | ATR-FTIR | ratio | 0.15 | 0.14 | 0.002 |
| Mineral strain | Strain | XRD | % | 0.69 | 0.66 | 0.01 |
| Mineral Size | Size | XRD | nm | 32.31 | 31.95 | 0.32 |
| Coherence length at 002 peak | CL002 | XRD | nm | 23.73 | 23.73 | 0.08 |
| Coherence length at 004 peak | CL004 | XRD | nm | 18.83 | 18.77 | 0.1 |
| Coherence length at 030 peak | CL030 | XRD | nm | 8.23 | 8.26 | 0.04 |
| Coherence length at 210 peak | CL210 | XRD | nm | 9.7 | 9.7 | 0.01 |
| Length of crystal cell | ‘a’axis | XRD | Å | 9.42 | 9.42 | 0.0003 |
| Length of crystal cell | ‘c’axis | XRD | Å | 6.9 | 6.9 | 0.0003 |

**Supplementary Table 2.** Physicochemical and histomorphometric parameters. Abbreviations, units, descriptive statistics and list of experimental methods for data acquisition (SD: standard deviation).


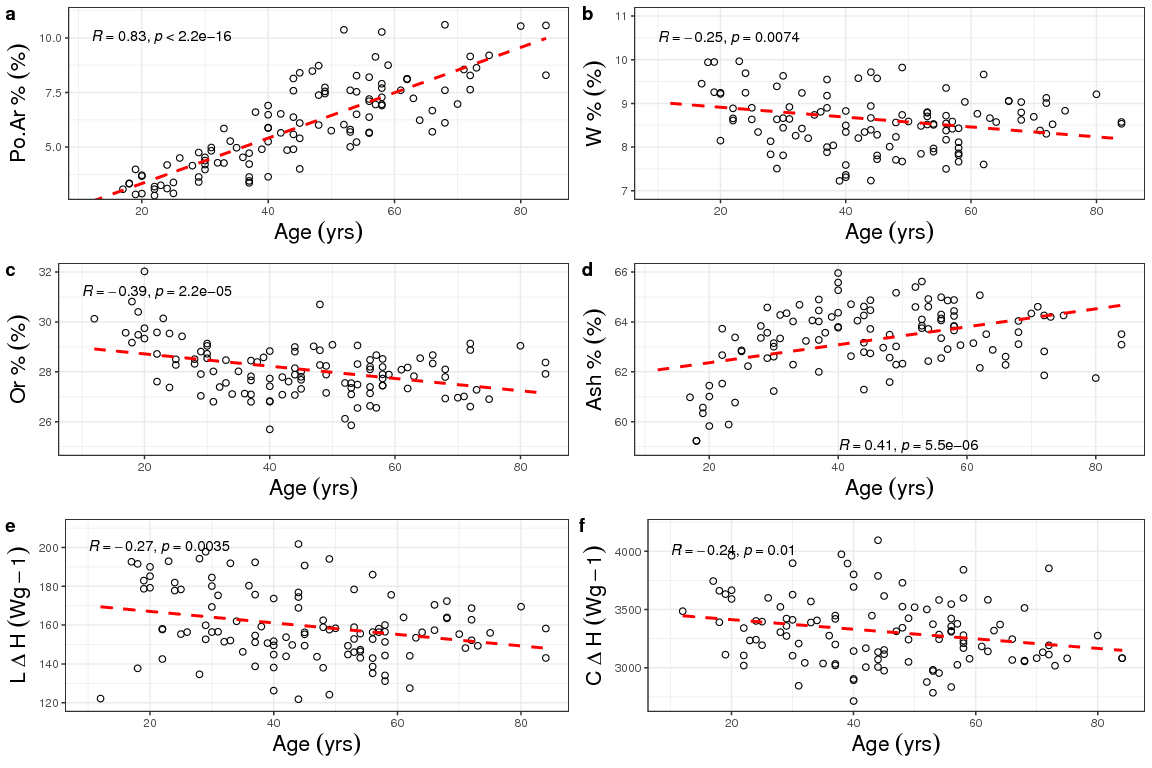
**Supplementary Figure 1.** Scatterplots of the relationship between thermal analysis and (b-f) and optical porosity (a) and age reporting correlation coefficient and extended p-value.


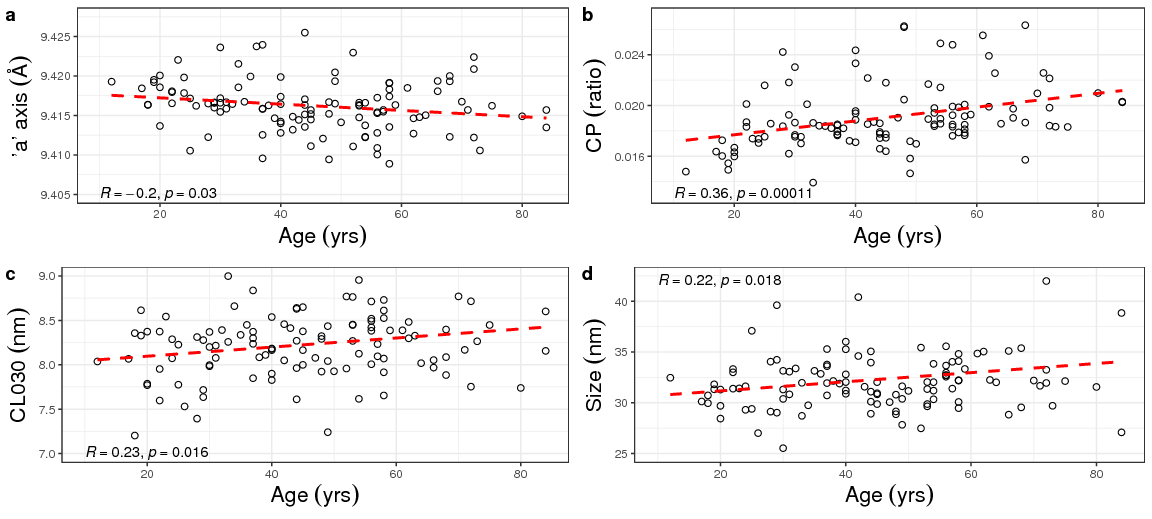


**Supplementary Figure 2**. Scatterplots of the relationship lattice cell size along the ‘a’axis (a), carbonate content (b), coherence length at 030 peak (c) and crystallite size obtained by W-H plot (d) with age reporting correlation coefficient and extended p-value.


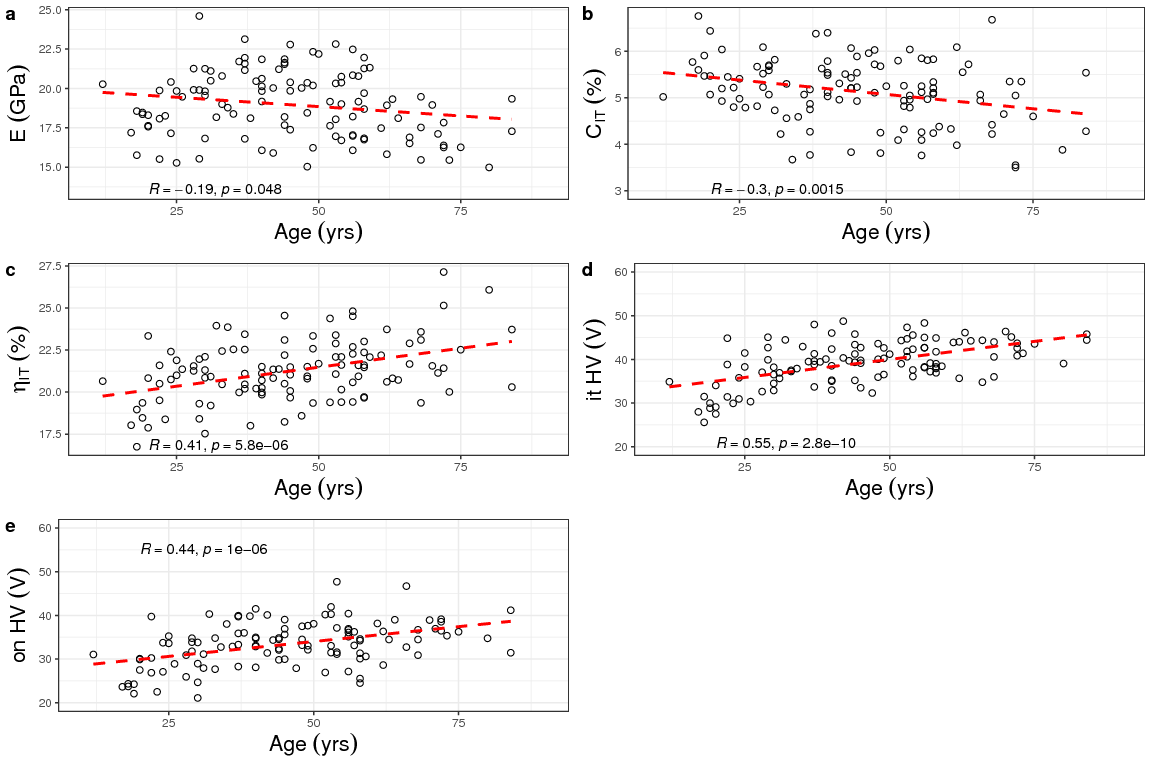


**Supplementary Figure 3.** Scatterplots of the mean tissue values for mechanical properties (a to c) with age reporting correlation coefficient and extended p-value. The last two plots report results for interstitial (d) and osteonal (e) microhardness.


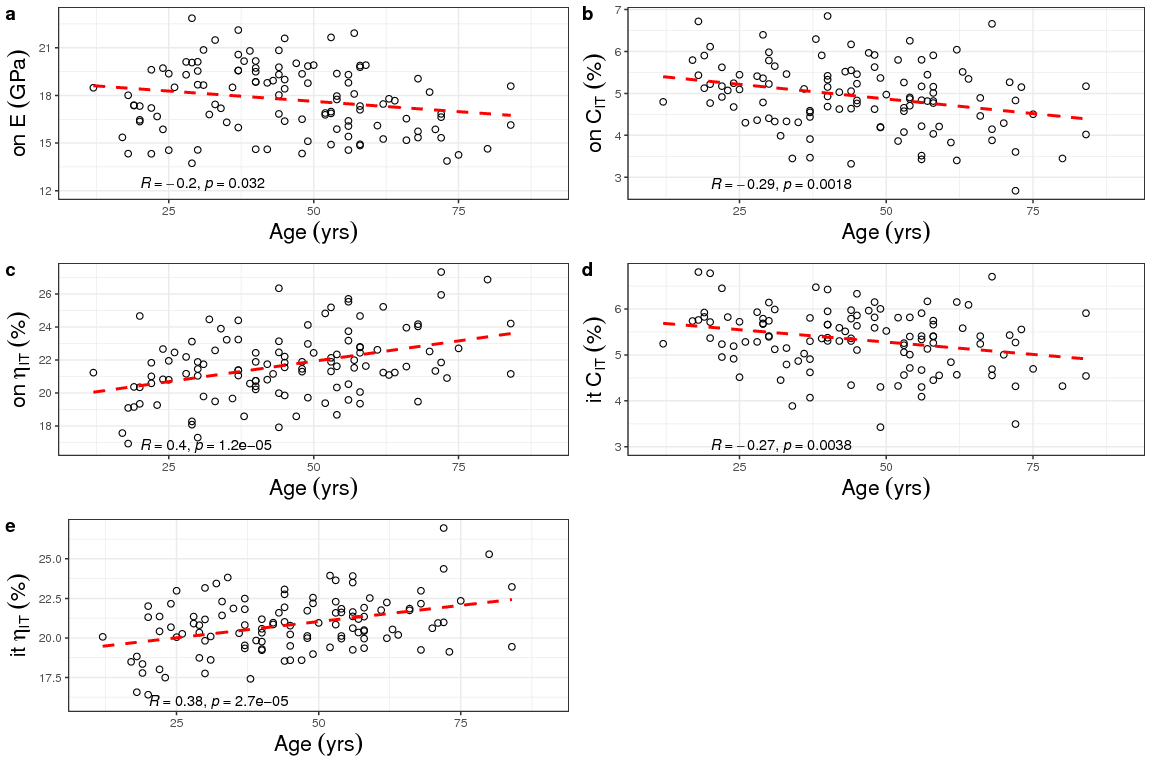


**Supplementary Figure 4.** Scatterplots of the mechanical properties of osteonal (a-c) and interstitial area (d, e) with age reporting correlation coefficient and extended p-value.
